# Biosignal-Based Attention Monitoring to Support Nuclear Operator Safety-Relevant Tasks

**DOI:** 10.3389/fncom.2020.596531

**Published:** 2020-12-21

**Authors:** Jung Hwan Kim, Chul Min Kim, Eun-Soo Jung, Man-Sung Yim

**Affiliations:** ^1^Department of Nuclear and Quantum Engineering, Korea Advanced Institute of Science and Technology, Daejeon, South Korea; ^2^Technology Research, Samsung SDS, Seoul, South Korea

**Keywords:** electroencephalography, eye movements, machine learning, attention, human error, nuclear safety

## Abstract

In the main control room (MCR) of a nuclear power plant (NPP), the quality of an operator's performance can depend on their level of attention to the task. Insufficient operator attention accounted for more than 26% of the total causes of human errors and is the highest category for errors. It is therefore necessary to check whether operators are sufficiently attentive either as supervisors or peers during reactor operation. Recently, digital control technologies have been introduced to the operating environment of an NPP MCR. These upgrades are expected to enhance plant and operator performance. At the same time, because personal computers are used in the advanced MCR, the operators perform more cognitive works than physical work. However, operators may not consciously check fellow operators' attention in this environment indicating potentially higher importance of the role of operator attention. Therefore, remote measurement of an operator's attention in real time would be a useful tool, providing feedback to supervisors. The objective of this study is to investigate the development of quantitative indicators that can identify an operator's attention, to diagnose or detect a lack of operator attention thus preventing potential human errors in advanced MCRs. To establish a robust baseline of operator attention, this study used two of the widely used biosignals: electroencephalography (EEG) and eye movement. We designed an experiment to collect EEG and eye movements of the subjects who were monitoring and diagnosing nuclear operator safety-relevant tasks. There was a statistically significant difference between biosignals with and without appropriate attention. Furthermore, an average classification accuracy of about 90% was obtained by the k-nearest neighbors and support vector machine classifiers with a few EEG and eye movements features. Potential applications of EEG and eye movement measures in monitoring and diagnosis tasks in an NPP MCR are also discussed.

## 1. Introduction

Attention is an important cognitive resource for information processing directly affecting the quality of task performance (Wickens et al., 1998). According to the Nuclear Event Evaluation Database (NEED), a database developed by Korea Institute of Nuclear Safety (KINS), approximately 20% of the unplanned nuclear power plant (NPP) shutdowns between 2000 and 2011 in Korea were due to human errors (Lee et al., 2017). The operator's insufficient attention accounted for more than 26% of the total cause of human errors, which takes the biggest portion. Hence, the decreased attention of an NPP main control room (MCR) operator could lead to a decrease in their situational awareness, which could result in a poor reactor operating performance and ultimately cause critical human errors.

Recently developed NPP designs include fully digitalized instrumentation and control (I&C). These upgrades are expected to enhance plant and operator performance. Advanced MCRs based on digital I&C technology create a completely different operating environment from the existing MCR configurations (Choi et al., 2019).

MCR operators are required to monitor several information sources, such as indicators, alarms, controllers, and mimic displays, but they have a limited capacity of attention (Wickens et al., 1998; Ha et al., 2016). Selective attention to important information is therefore required to effectively understand the current reactor operating status (Mumaw et al., 2000). MCR operators therefore allocate their attention resources and selectively pay attention to relevant and important information to understand the system status.

MCR operators' tasks involve cognitive activities of monitoring and detecting the environment, diagnosing situations, and decision making (Yang et al., 2017; Kim and Seong, 2019). MCR operators generally monitor the plant status and diagnose and respond to the plant status for abnormal operation (Kim et al., 2020b).

There is a direct relationship between operation and attention, and that is why either a supervisor or peers observe other operators to check whether they are sufficiently attentive. However, this method requires significant labor and may be subjective. The problem could be exacerbated in an advanced MCR, where personal computer-based workstations make it difficult for a supervisor to detect fellow operators' attention states (Savchenko et al., 2017). In this situation, remote measurement of attention would be a useful tool providing real-time feedback to the supervisor.

Recently, the analysis of electroencephalography (EEG) and eye movements have been used to assess variations in the attention state of subjects during the execution of cognitive tasks in various fields (Jung et al., 2017, 2019; Kim et al., 2018; Pei et al., 2018).

Liu et al. (2013) determined whether students remain attentive throughout instruction during the learning process based on their EEG signals. To describe the learning environment, Standard English class material was used as experiment material. A classification accuracy of 76% was obtained through the support vector machine. The authors explained that if teachers identify whether students are attentive, they can remind students to remain focused, thereby improving students' learning effects.

Heuer and Hallowell (2015) suggested the eye movement method to index attention allocation in people with aphasia. Auditory sentence comprehension and visual search tasks were performed. The authors observed differences in attention allocation between groups with and without aphasia depends on task complexity in single- and dual-task conditions. They suggested that utilizing information from eye movements has promising potential for clinical assessment applications.

Pallavi and Harish (2016) implemented a driver's attention monitoring system using EEG signals. The EEG signals were monitored and analyzed by using a brain sense headband that transmits the information to the controller wirelessly using the Bluetooth module. The warning tone would be triggered to prevent accidents when the drowsiness condition occurred to the driver. The authors explained that their EEG based-attention monitoring system can be used to indicate driving attention and drowsiness.

To date, the potential benefits of studying EEG and eye movements together to understand operator's attention in NPP tasks has not been pursued. As MCR operators perform cognitive activities by using information obtained through visual channels, we evaluated the use of both EEG signals and eye movements (as supportive biosignal) to establish a robust baseline to determine the plausibility of developing an attention monitoring system in this paper. Using two sets of data to monitor human attention may help to improve the accuracy in model predictions and contribute to overall human error reduction.

In this research, performing nuclear tasks based on the use of a nuclear simulator was investigated. Although this is not completely the same as with the tasks of a professional MCR operator in NPP, this could provide similar environment of an MCR operator and raise the level of psychological involvement of the subjects during the experiments. To reflect and mimic operations in an advanced MCR, this study designed general tasks and nuclear simulator tasks as the basis for collecting relevant EEG and eye movement data. The collected data were analyzed for feature extraction and classification model development based on the use of machine learning algorithms.

## 2. Methods

This study constructed a hypothesis to investigate the identification of the attention of advanced MCR operators. The hypothesis is that there will be a significant difference in an operator's biosignals between the presence and absence of attention while performing general tasks as well as the tasks related to nuclear reactor operations.

### 2.1. Experimental Design

To test this hypothesis, study subjects were asked 38 questions as general tasks and 72 questions specific to nuclear reactor operations based on the use of the nuclear simulator. EEG and eye movement data were collected during the experimental sessions and analyzed with respect to the testing hypothesis.

#### 2.1.1. General Tasks

**Figure 1:**
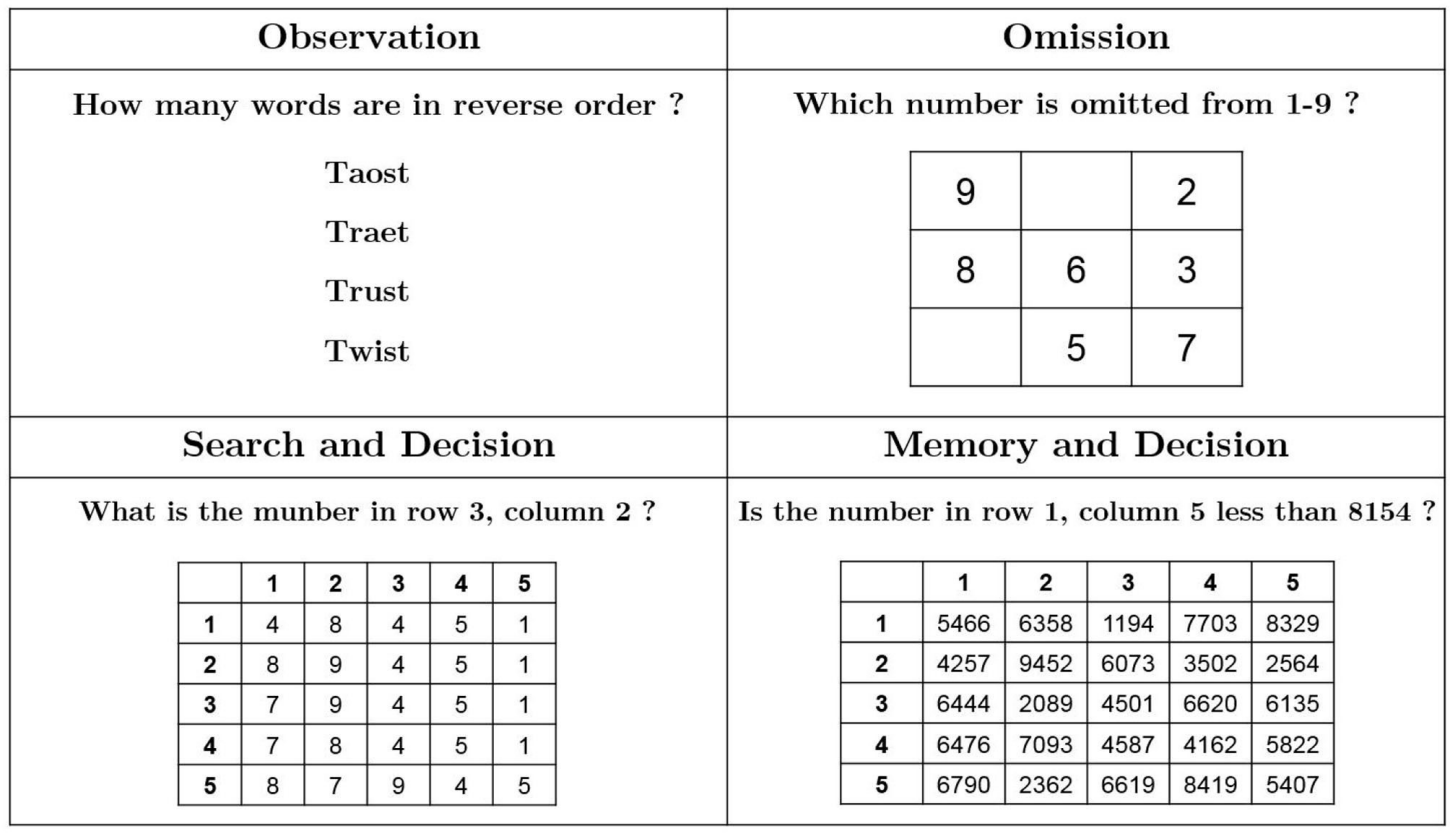
Examples of general tasks.

Oh and Lee (2013) investigated the potential causes of human errors in an advanced MCR where PC-soft controls are heavily relied on for reactor operation. They found that observation, omission, search and decision, and memory and decision failures are four major factors related to human errors. The authors also designed four experimental tasks to investigate the role of these factors. These tasks were used with slight modification in the current study as general task questions. Examples of these questions are illustrated in Figure 1. For example, the observation trial questions ask the subject how many words in a list (“taost,” “traet,” “trust,” “twist”) have typographical errors [answer: two words (toast and treat) have typographical errors]. The omission trial questions ask the subject to find the omitted numbers from a matrix where the numbers between 1 and 9 are presented in a random order. The search and decision trial questions ask the subject to check the number in a specific position in a 5X5 matrix. The memory and decision trial questions ask the subject to recall a specified number in a 5X5 matrix and compare it with the number provided. A total of 38 questions were used in the general task session, including eight observation trials, six omission trials, 16 search and decision trials, and eight memory and decision trials.

**Figure 2:**
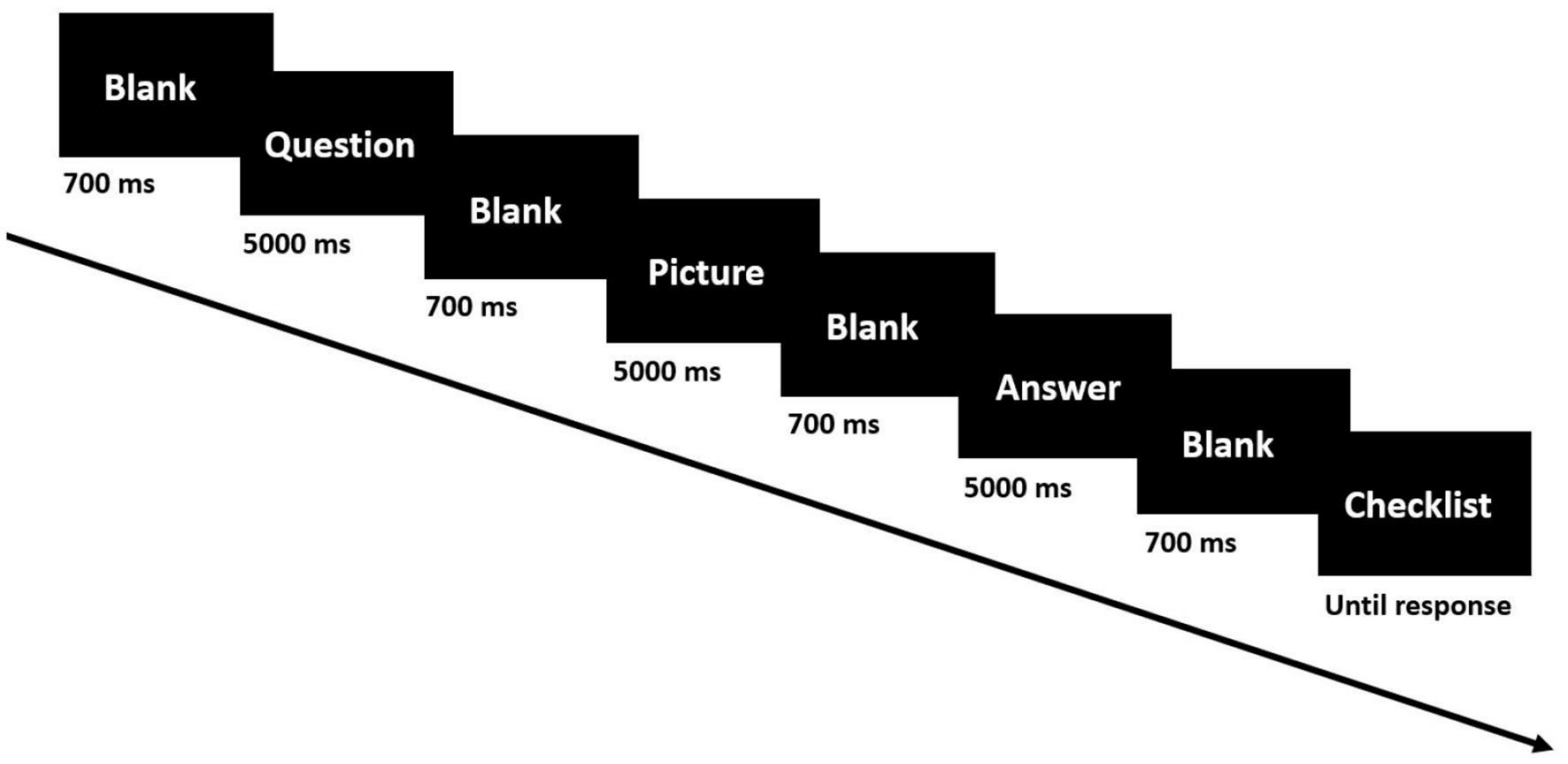
Experiment paradigm of general tasks.

As shown in Figure 2, each trial followed the same sequence of screen changes: a fixation cross to prepare the subject for the trial, a blank screen, a question (e.g., “What is the number in row 3, column 5?”), a blank screen, a picture related to the question (e.g., a matrix of numbers with 5 rows and 5 columns), a blank screen, an answer to the question provided by the instructor (in each trial, the subjects answered the trial questions and were given an opportunity to compare their answer with the answer provided by the instructor.), and a blank screen followed by a two checklist questions to be answered by the subject via keyboard. The first checklist question was “Was there an error in the instructor's answer?” with the choice of yes or no. The second question was “How attentive were you in answering the previous question?” with these multiple-choice options: very attentive, moderately attentive, somewhat attentive, only slightly attentive, or not at all attentive.

#### 2.1.2. Nuclear Simulator Tasks

The second group of trials used soft controls in an advanced MCR mock-up called the Windows-based Nuclear Plant Performance Analyzer (Win-NPA). Win-NPA is a compact nuclear simulator capable of simulating 53 malfunctions in nuclear reactor operations (Kim et al., 2000; Sohn et al., 2011). Although the simulator is not a full scope simulator, many researchers have used it to simulate operations in an advanced MCR (Choi et al., 2018; Kim et al., 2020c). The interface of the Win-NPA is fully digitalized to make the experimental environment similar to the environment in an advanced MCR.

Various types of human error can occur in an advanced NPP MCR. This study examines operation omission, wrong object selection, and wrong operation as part of conducting nuclear simulator tasks (Kim et al., in press). Operation omission can be defined as failing to execute a step in an operating procedure (e.g., mistakenly taking steps 1, 2, 4, and 5 in a procedure, leaving out step 3). Wrong object selection is a failure to select a target object and instead select a different object. An example of the wrong operation is pressing an “OPEN” button instead of a “CLOSE” button.

The types of scenarios used in the experiment in association with identifying human error occurrence were normal operating scenarios (i.e., startup and shutdown) as well as two accident scenarios [i.e., a loss of coolant accident (LOCA) and a steam generator tube rupture (SGTR)]. If a LOCA occurs in an NPP, the pressurizer's (PZR's) pressure, temperature, and the water level will decrease, and containment radiation will increase. Similarly, if an SGTR occurs in an NPP, the PZR's pressure, temperature, and the water level will decrease, and the steam generator (SG) water level will increase. Prior to the experiment, the subjects were instructed that it was training session for education to avoid the higher workload or decision burden for accident scenarios. To focus on evaluating the attention of the subjects, the scenarios used in the experiment was not required to recover the accident but was required to monitor and diagnose the situation.

**Figure 3:**
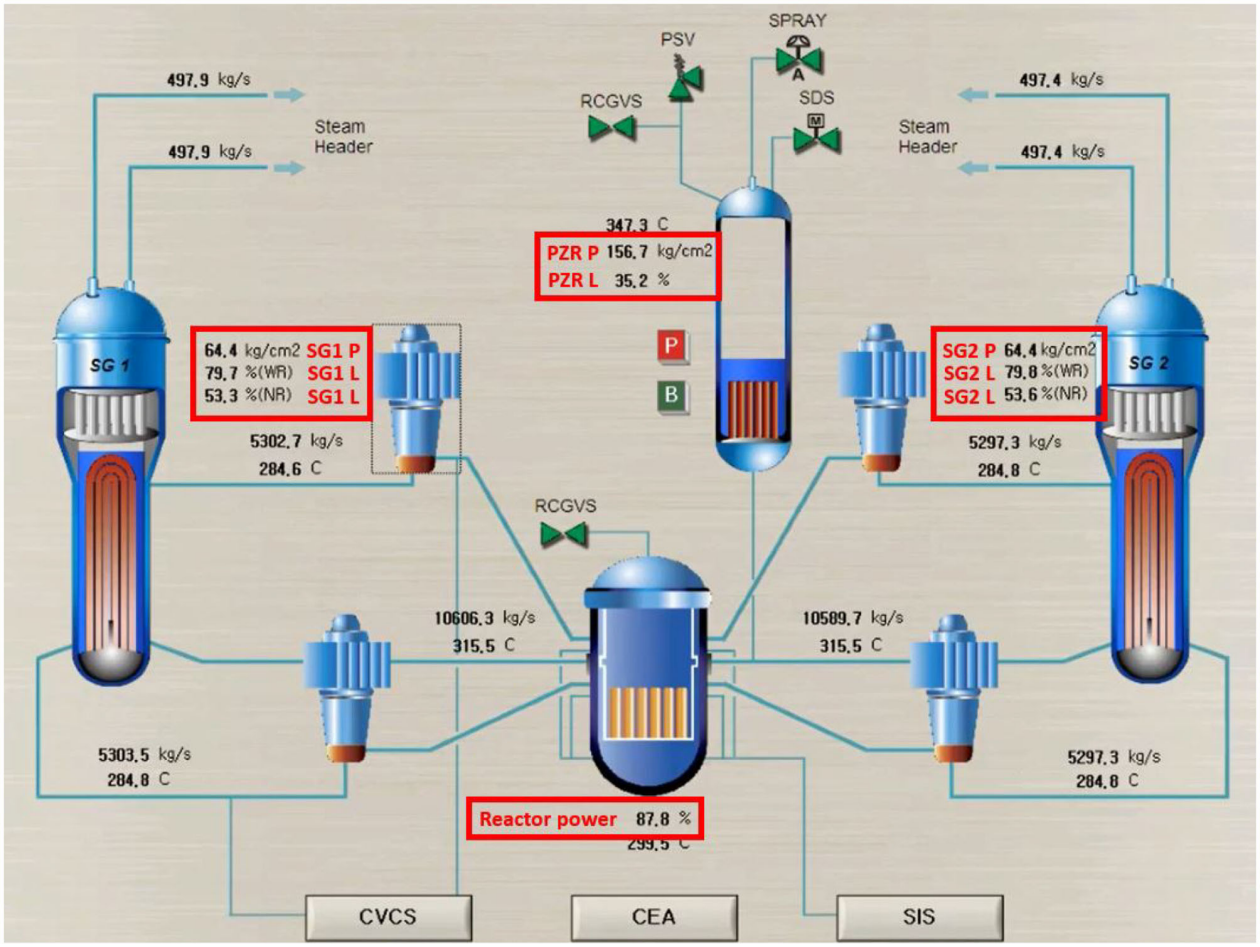
Schematic diagram of nuclear simulator tasks with four groups of AOI.

To analyze the cognitive behavior of the subjects during the trials, this study defined four groups of areas of interest (AOI, or specific operating parameters of interest) as shown in the red rectangles of Figure 3; these are (1) pressure, wide-range water level, and narrow-range water level of SG1, (2) pressure, wide-range water level, and narrow-range water level of SG2, (3) PZR pressure and water level, and (4) reactor power. Furthermore, there is an additional condition that insert a broken indicator (BI) to make deviations from the training contents. While faced with a scenario (normal operation, LOCA, or SGTR), the subject must focus on the four groups of AOI to monitor indicators or find a BI. Collectively, this study provides 18 startup scenarios, 18 shutdown scenarios, 18 LOCA scenarios, and 18 SGTR with each having six scenarios of finding BIs.

**Figure 4:**
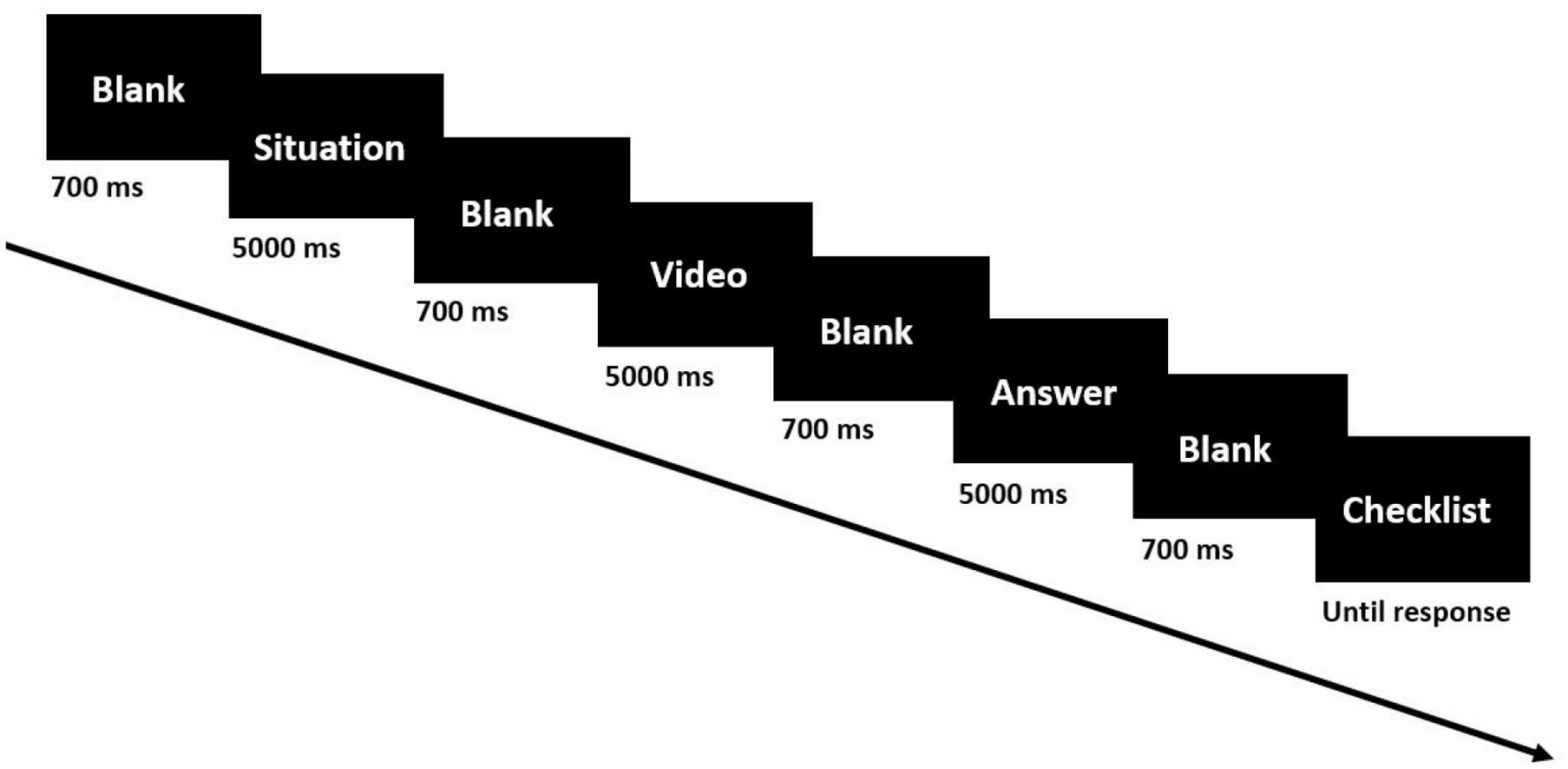
Experiment paradigm of nuclear simulator tasks.

Each subject acted as an operator, specifically a reactor operator or a turbine operator. Each subject took part in a session of 72 operator action trials, 36 involving normal scenarios, and 36 involving the two accident scenarios. As shown in Figure 4, each trial followed this screen sequence: a fixation cross to prepare the subject for the trial, a blank screen, a question (e.g., “Can you find a broken indicator?”), a blank screen, a video related to the question (e.g., a video of the LOCA scenario), a blank screen, the instructor's answer [e.g., PZR pressure indicator is broken (PZR P is BI)], a blank screen, a checklist with two questions to be answered by the subject via keyboard. As in the case of the general task session, the first checklist question was “Was there an error in the supervisor's answer?” with the choice of yes or no. The second question was “How attentive were you in answering the previous question?” with these multiple-choice options: very attentive, moderately attentive, somewhat attentive, only slightly attentive, or not at all attentive.

As the study requires labeling of the experimental data as either presence of attention (PoA) or absence of attention (AoA) for classification model development, the answers to the checklist questions were used to identify the attention levels of the subjects during the experimental sessions.

### 2.2. Subjects of the Experiment

Because the tasks performed in this study require sufficient knowledge in nuclear reactor systems, the experimental subjects were recruited among the college/graduate students majoring in nuclear engineering. It was required for the subjects to have completed one of the two courses: “Introduction to Nuclear Engineering” or “System Engineering of Nuclear Power Plants.” In the end, 30 volunteer students (27 male and three female) from the Department of Nuclear and Quantum Engineering at Korea Advanced Institute of Science and Technology (KAIST) participated in the experiments. They were also screened against a history of eye problems, neurological disorders, mental disorders, or alcohol or drug dependence. None of the students were disqualified from the screening.

Before conducting the experimental sessions, the research staff explained the experimental procedures to the subjects, as required by the KAIST Institutional Review Board (IRB) guideline. The subjects read an information sheet and signed an agreement regarding the data collection process. All subjects were required to sleep more than 6 h and not to drink caffeine or alcohol for at least 24 h before the experiment.

Prior to performing the experiment, there was a 30 min training session by the experiment instructors, who have considerable expertise with the Win-NPA system. This training session included conducting simple tasks to show how to monitor and diagnose simulator scenarios. The actual experiment was conducted only for the subjects who answered more than six out of eight questions correctly in the pre-test. It turned out that one student did not pass the pre-test. After further studies, the student was qualified and participated in the experiment.

### 2.3. System Architecture for Classification

#### 2.3.1. Data Acquisition

Studies indicate that among various frequency bands of EEG, an increase in the gamma band and a decrease in the alpha band are associated with subjects' paying attention to tasks (Pascucci et al., 2018). In eye movements, an increase in the fixation count and fixation duration is often referred to as an increase in the attention level (Holmqvist et al., 2011). Based on these observations, we acquired the relevant EEG and eye movement data to evaluate the attention status of the subjects.

**Figure 5:**
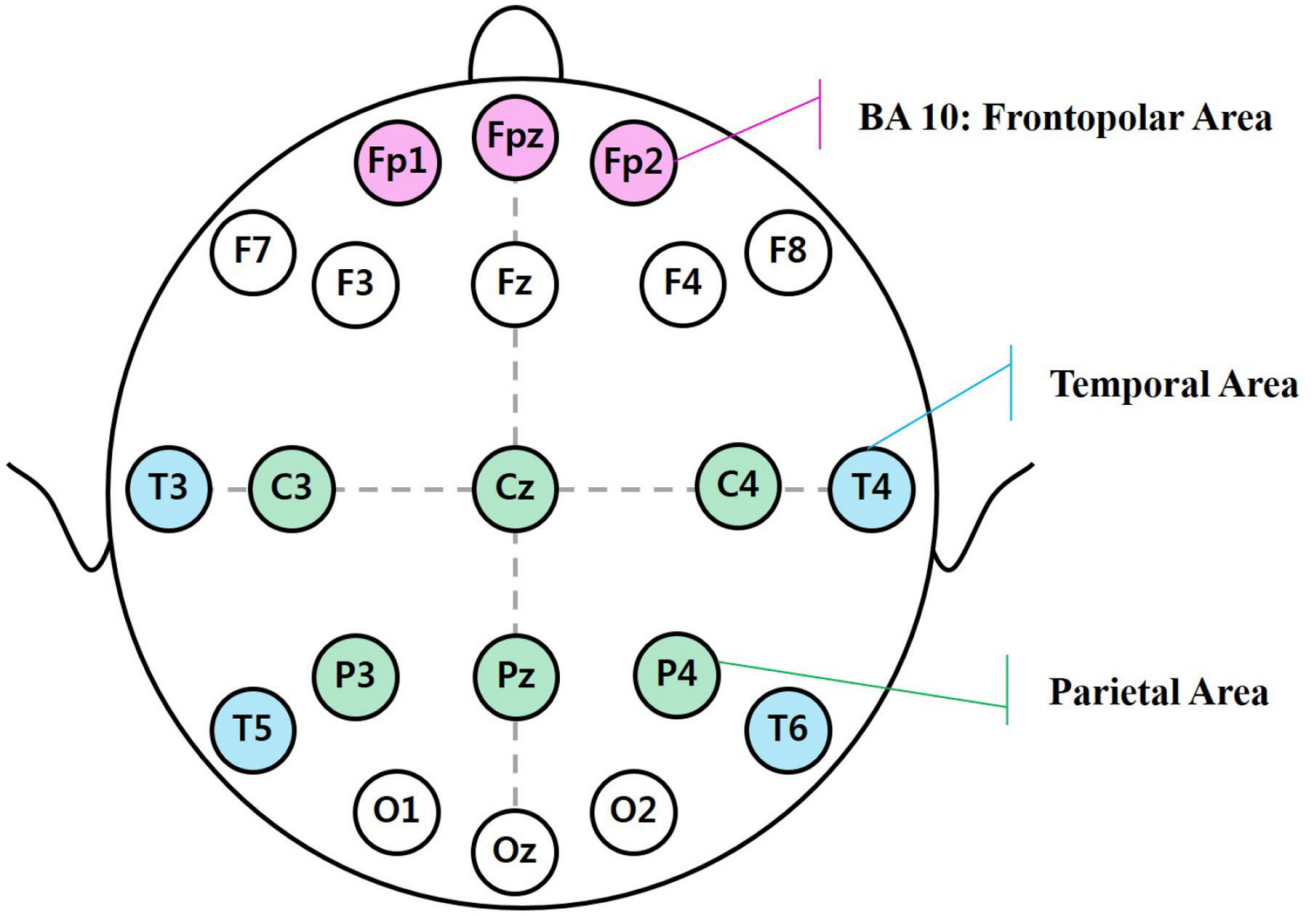
Brain areas corresponding to the 10/20 electrode positions.

EEG signals were measured using a Neuron-spectrum 4/P (Neurosoft Ltd., Russia). Each subject was fitted with an Ag/AgCl electrode cap arranged with an extended international 10-20 system. The EEG data from 21 channels (Fp1, Fp2, Fpz, F3, F4, F7, F8, Fz, C3, C4, Cz, T3, T4, T5, T6, P3, P4, Pz, O1, O2, and Oz) were recorded, as shown in Figure 5, at a sampling rate of 500 Hz. Reference electrodes were placed on both earlobes. During the experiment, the electrode impedances of all the channels were kept below 5 kΩ.

The eye movements were measured by using a Tobii X120 eye tracker. The eye tracker was located beneath the computer monitor, monitoring the subject's field of vision. The seating position was adjusted according to the subject's height. Since the eye tracker is non-invasive and operates remotely, the device did not interfere with the subject's task performance.

After adjusting the seating position, the Tobii X120 was calibrated. Calibration required the subjects to move their eyes to five specific spots, i.e., each of the four corners and the center of the monitor screen. These eye movements were tracked using a standard five-point calibration option in the Tobii Eye Tracker Extension for Presentation software. Eye movement was recorded with a sampling frequency of 120 Hz. Because EEG and eye movement data collection are sensitive to light and sound, the experimental environment blocked light and sound to support the subject's concentration. The EEG and eye movement data were synchronized in time by using the Neurobehavioral Systems presentation software.

#### 2.3.2. Channel Selection

The EEG signals are generally categorized as delta (δ), theta (θ), alpha (α), beta (β), and gamma (γ) based on signal frequencies (Zeng et al., 2015). The δ frequency (1–4 Hz) appears in cognitive processes related to the detection of salient stimuli in the environment. The θ frequency (4–8 Hz) is related to visual selective attention. The α frequency (8–13 Hz) primarily reflects visual processing in the brain (Klimesch et al., 2011). The β frequency (13–30 Hz) focuses on neural correlates of attention and concentration (Wang et al., 2017). The γ frequency (30–50 Hz) reflects working memory and attention (Klimesch et al., 2008). These EEG indicators were measured on various parts of the brain to represent different brain functions. This study focused on describing functions related to the frontal, temporal, and parietal lobes as well as the Brodmann Areas (BAs).

As shown in Figure 5, each area of the brain is responsible for a specific function. The frontal lobes play a role in many processes, such as motivation, intention, attention, and concentration. The temporal lobes are believed to be of central importance in memory processing and discrimination of complex visual stimuli (Klimesch et al., 1994). The parietal lobes are associated with the detection of salient new events in the environment and in sustaining attention on task goals. BA 10 is the frontopolar area responsible for central executive processes such as memory, emotion, and integration of the information (Peng et al., 2018).

The brain areas that correspond to the 10/20 electrode positions can vary and include the frontal area (with Fp1, Fp2, Fpz, F3, F4, F7, F8, Fz, C3, C4, and Cz), the temporal area (with T3, T4, T5, and T6), the parietal area (with C3, C4, Cz, P3, P4, and Pz), and the BA 10 area (with Fp1, Fp2, and Fpz).

#### 2.3.3. EEG and Eye Movement Preprocessing

To remove artifacts in the collected EEG data, data preprocessing was performed based on Makoto's preprocessing pipeline using EEGLAB (Delorme and Makeig, 2004). The line noise was removed by the CleanLine plugin (Mullen, 2012). Bad channels were rejected using the Clean Rawdata plugin, and continuous data were corrected using artifact subspace reconstruction (ASR). The Adaptive Mixture Independent Component Analysis (AMICA) program and the postAmicaUtility toolbox were used for independent component analysis (ICA) (Palmer et al., 2012). The artifacts from body movement, rolling eyeballs, and blinking were excluded from the analysis based on visual inspections of each component.

The preprocessed data were divided into 38 epochs of 5 s for each general task and 72 epochs of 5 s for each nuclear simulator task. A total of 110 epochs of 5 s per channel were collected and used for subsequent analysis.

The eye movement data are made up of x, y coordinates with each data point's associated timestamp. These raw data were used to obtain information about fixation count and fixation duration. Preprocessing of eye movement data includes conversion of gaze position data from pixels to millimeters, removal of blinks and artifacts, and removal of outliers.

#### 2.3.4. Feature Extraction

To support the development of a machine learning algorithm, this study extracted a set of features that describe subjects' EEG and eye movement responses. These features were then used to classify the presence and absence of attention using the classification model. The EEG features used were extracted from the frequency domain (Tang et al., 2013).

The frequency domain features were calculated using the Discrete Fourier Transform (DFT). The transformed data were categorized into five frequency bands, δ, θ, α, β, and γ. A relative power value was calculated for each channel by dividing the power of each frequency band by the total power from the five frequency bands.

In the case of eye movement data, fixation was defined as a pause, of 150 ms, in eye movement over a specific region of the visual field. In the nuclear simulator tasks, a question was asked referring to four groups of AOI (SG1, SG2, PZR, and reactor power). All fixations occurring during the 5 s video relevant to the question were measured but only the fixations on specific group of AOI were considered for analysis. If the subject looked at other groups of AOI, these fixations were not included in the analysis. The analysis consisted of calculating the total number of fixations on the relevant group of AOI and the time duration of these fixations per task.

This study used each of the five frequency domain features from each channel of the EEG measurements and two features extracted from the eye movement data as summarized in Table 1.

**Table 1:** Features extracted from the EEG and eye movement data.

**Biosignals**	**Feature types**	**Extracted features**
EEG	Frequency domain	Relative power of δ, θ, α, β, and γ
Eye movements	Fixation domain	Total number of fixations in the relevant AOI (fixation) and total time spent on the relevant AOI fixation (duration)

## 3. Results

### 3.1. Statistical Analysis

Using the answers to the two checklist questions in both the general and nuclear simulator task sessions, this study labeled the collected EEG signals and eye movement data into the PoA class and the AoA class. Trials with the answer “very attentive” and “moderately attentive” with correct responses for the task were labeled as the PoA class, and those with “somewhat attentive” were not used in the classification. The AoA class was defined for the following two conditions. First, the trials with the answer “only slightly attentive” or “not at all attentive” in the second question of the checklist were labeled as the AoA class regardless of the correctness of the answer to the first question. Also, the cases with incorrect responses to the first question of the checklist were also labeled as the AoA class by assuming that the wrong answer was due to a lack of attention. This may involve misclassification as the subject could have answered the question wrong under full attention. But the number of cases under this category was very small and is not expected to affect the outcome of the study. In summary, there were 2656 PoA cases, 578 AoA cases, 12 cases of potential misclassification, and 54 removed cases due to artifact removal. To examine the effect of possible misclassification with the 12 cases, this set was treated as both the AoA class and the PoA class in the analysis. The results showed statistically insignificant difference with and without these 12 cases both in the analysis of the general tasks and the nuclear simulator tasks.

**Figure 6:**
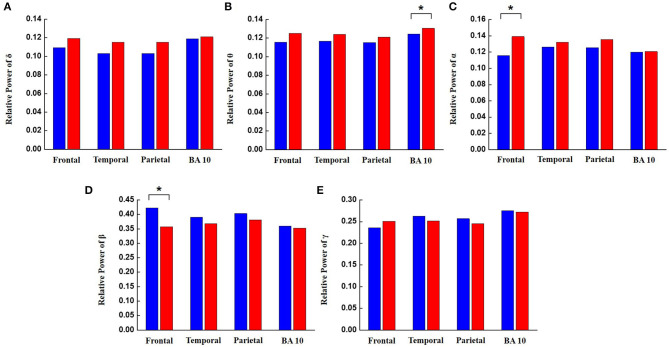
The relative power of EEG frequency bands between the AoA class and the PoA class in different regions of the brain for all general tasks. Blue bars represent the PoA class and the red bars represent the AoA class. Brain areas with *P*-values less than 0.05 are identified with an asterisk. **(A)** Relative power of delta, **(B)** relative power of theta, **(C)** relative power of alpha, **(D)** relative power of beta, and **(E)** relative power of gamma.

The results from the Welch's *t*-test for the general task questions are shown in Table 2. Results indicated that statistically significant differences exist in several of the EEG signals between the AoA class and the PoA class. These EEG signals were the relative power of the α and β bands from the frontal lobes and the relative power of the θ band from the BA 10. As mentioned above, frontal lobes are related to attention and concentration, and BA 10 is responsible for memory and integration of information. The α, β, and θ bands are related to visual processing, concentration, and visual selective attention, respectively. As shown in Figure 6, the θ band was significantly increased across the BA 10 in the AoA class. Also, the α band was significantly increased and the β band was significantly decreased across frontal lobes in the AoA class.

**Table 2:** *P*-values for the EEG indicators showing differences between the AoA class and the PoA class for all general tasks while viewing pictures.

**Indicator**	**Frontal**	**Temporal**	**Parietal**	**BA 10**
Relative power of δ	0.633	0.587	0.545	0.535
Relative power of θ	0.420	0.597	0.606	0.011*
Relative power of α	0.008*	0.521	0.242	0.587
Relative power of β	0.010*	0.229	0.304	0.143
Relative power of γ	0.372	0.521	0.457	0.592

*P-values less than 0.05 are identified with an asterisk*.

**Figure 7:**
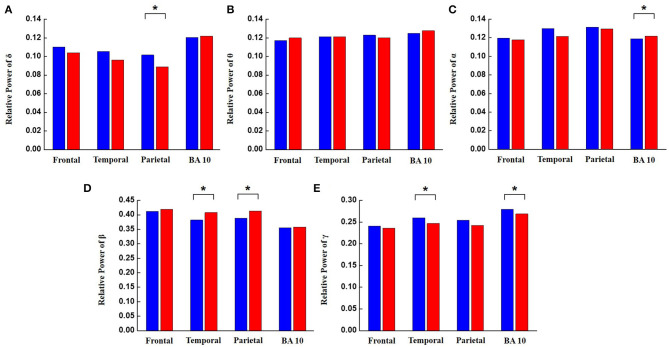
The relative power of EEG frequency bands between the AoA class and the PoA class in different regions of the brain for all Win-NPA tasks. Blue bars represent the PoA class and the red bars represent the AoA class. Brain areas with *P*-values less than 0.05 are identified with an asterisk. **(A)** Relative power of delta, **(B)** relative power of theta, **(C)** relative power of alpha, **(D)** relative power of beta, and **(E)** relative power of gamma.

The EEG signals collected during the Win-NPA tasks also showed significant differences (based on the Welch's *t*-test) between the AoA class and the PoA class as shown in Table 3. Because visual processing of information is important cognitive activities during the nuclear simulator session, EEG signals recorded from the brain regions related to visual processing, such as on temporal lobes and parietal lobes, showed significant differences between the AoA class and the PoA class. Also, EEG signals from BA 10, which is associated with memory and integration of information showed a significant difference between the AoA class and the PoA class, similar to the observations from the general tasks. As shown in Figure 7, the δ band was significantly decreased across parietal in the AoA class while watching the videos. The α band was significantly increased across BA 10 in the AoA class. This result is consistent with previous studies that alpha desynchromization promotes information processing in the brain (Klimesch, 2012). Similarly, the β band was significantly increased across temporal and parietal lobes in the AoA class. The γ band was significantly decreased across temporal and BA 10 in the AoA class.

**Table 3:** *P*-values for the EEG indicators showing differences between the AoA class and the PoA class for all Win-NPA tasks while watching videos.

**Indicator**	**Frontal**	**Temporal**	**Parietal**	**BA 10**
Relative power of δ	0.214	0.051	0.036*	0.546
Relative power of θ	0.576	0.995	0.638	0.131
Relative power of α	0.662	0.051	0.619	0.039*
Relative power of β	0.598	0.003*	0.041*	0.462
Relative power of γ	0.443	0.015*	0.057	0.019*

*P-values less than 0.05 are identified with an asterisk*.

As shown in Tables 2, 3, the number of statistically significant indicators was three in the general tasks and six in the nuclear simulator tasks. The fact that the nuclear simulator tasks required professional knowledge and higher concentration levels compared to the general tasks may have resulted in a larger number of significant indicators between the AoA class and the PoA class in the nuclear simulator tasks. Additionally, it is noticeable that, unlike the general tasks, the nuclear simulator tasks showed statistically significant differences in the EEG signals from the temporal and parietal lobes, which are related to visual attention.

The observed differences in the attention level between the AoA class and the PoA class during the general tasks and nuclear simulator tasks were utilized for classification model development by applying machine learning algorithms.

### 3.2. Classification

Feature selection is the process of selecting a subset of features that contribute the most to the construction of the classification model through including and excluding features present in the data. The feature selection was achieved by using the default parameters of the Variable Selection using Random Forests (varSelRF) technique (Diaz-Uriarte, 2007). The varSelRF uses both backwards variable elimination and selection based on the potentially highly correlated variables. As described in the feature extraction section, five features from the frequency domain in the EEG data and two features from eye movement data were used for classification.

To classify the data, both the classifiers of the k-nearest neighbors (kNN) and support vector machine (SVM) were used. Theses classifiers are widely used in various fields to classify EEG data (López-Gil et al., 2016). The kNN is a supervised learning algorithm for classifying objects based on the closest training data in the feature space. As a non-parametric method, it performs classification based on comparing testing data with training data. The SVM is a supervised learning algorithm and formulates a separating hyperplane. The method is applied to solve a quadratic optimization problem in the feature space. Kernel SVM finds the optimum hyperplane into a higher dimensional space, which ensures that the distance between margins is maximum. This study specifically used the radial basis function (RBF) kernel to project input vectors into a Gaussian space.

30% of the study observations were randomly selected and used as testing data, and 70% of the observations were used as training data for classification model development. The average classification accuracy using the developed model was calculated through the classification results of testing data.

Table 4 summarizes the results as classification accuracy of the developed model for the case of the general tasks. The classification accuracy was calculated as an average across the total brain, frontal lobes, and BA 10 based on statistical analysis. When the five EEG frequency domain features were used, the average classification accuracies of the kNN and SVM classifiers were 84.6–86.7 and 87.0–87.4%, respectively. From the results, it is noticeable that the average classification accuracy of using BA 10 data only is comparable to that of using the total brain and frontal lobes data, which use a greater number of channels.

**Table 4:** Average classification accuracy of the AoA class and the PoA class in the general tasks using the frequency domain features (Unit: %).

**Brain areas**	**kNN**	**SVM**
Total brain	85.8	87.4
Frontal lobes	86.7	87.0
BA 10	84.6	87.2

Table 5 summarizes the average classification accuracy of the developed model between the AoA class and the PoA class in the nuclear simulator tasks. The classification accuracy was calculated as an average across the total brain, temporal lobes, parietal lobes, and BA 10 based on statistical analysis. When the five EEG frequency domain features were used, the average classification accuracies of the kNN and SVM classifiers were 86.2–86.8 and 87.2–87.8%, respectively. From the results, it is also noticeable that the average classification accuracy of using the BA 10 data is comparable to that of using the data from the total brain, temporal lobes, and parietal lobes which use a larger number of channels. The BA 10 is related to visual processing and attention functions.

**Table 5:** Average classification accuracy of the AoA class and the PoA class in the nuclear simulator tasks using the frequency domain features (Unit: %).

**Brain areas**	**kNN**	**SVM**
Total brain	86.5	87.8
Temporal lobes	86.5	87.6
Parietal lobes	86.2	87.4
BA 10	86.8	87.2

The results again confirm that visual processing and attention play an important role in understanding visual-based nuclear relevant tasks. On the basis of statistical analysis and classification results from the general tasks and the nuclear simulator tasks, the null hypothesis is rejected.

This study also compared the average classification accuracy between the case of using only the EEG data and the case of using both the EEG and eye movement data. Table 6 summarizes the comparison of the average classification accuracy of the AoA class and the PoA class between the two cases in the nuclear simulator tasks. The average classification accuracy was calculated for the total brain area.

**Table 6:** Average classification accuracy in the total brain when using EEG only and when using EEG with eye movements in the nuclear simulator tasks using the frequency domain features (Unit: %).

**Biosignals**	**kNN**	**SVM**
EEG	86.5	87.8
EEG with eye movements	89.1	90.1

The average classification accuracies of the kNN and SVM classifiers were about 86-87% for the use of EEG data only, and 89–90% for the combined use of the EEG and eye movement data, respectively. The average classification accuracy of using both the EEG and eye movement data is about 3% higher than using only the EEG data. Although the increase is not great, these results may indicate the potential of the eye movement data as the supportive biosignal to evaluate MCR operators' attention.

## 4. Discussion

### 4.1. Applications

The proposed system could be utilized to provide a real-time monitoring of the attention levels of nuclear reactor operators during operations in the MCR. Such monitoring capability may help to enhance overall performance of the reactor operating team without interfering with their operating duties or functions. Such capability may also provide opportunities to prevent or detect human errors, particularly in terms of an advanced NPP MCR.

Applying the proposed concept in an NPP MCR requires a high degree of information security in data utilization. The process of obtaining and transmitting EEG and eye movement data should be protected to prevent tampering or unauthorized acquisition of the data. For this reason, application of the proposed system to an advanced MCR can be through wired data transmission in conjunction with a secure USB or using the Intranet or using one-way data transmission and reception (this is because Wi-Fi and Bluetooth tools are not allowed in NPPs).

Another application of the proposed approach is to support operator training. The operators' performance during training sessions can be monitored in real-time as suggested in the study. Based on the analysis of the data, individually tailored recommendations can be provided to the trainees conserving the privacy of the data. Also, effectiveness of the existing training programs can be assessed by using the proposed approaches for program enhancement.

### 4.2. Biosignal and Channel Selection

In this study, use of all the available EEG channels and their potentially associated features were not considered under the consideration of avoiding overfitting of the machine learning algorithm. In fact, taking economic and ergonomic aspects into consideration, recording a full set of EEG data may not be desirable (Kim et al., 2020a). To examine this point, this study compared the EEG results from the total brain (21 channels) to the BA 10 (three channels: Fp1, Fp2, and Fpz).

Comparison of the classification accuracies from using various brain channel data indicated that using all available data or channels was not necessary for the given task, i.e., classification of attention levels. As shown in Table 5, the average classification accuracies of using only the BA 10 data are comparable to those of using the total brain data. This suggests that an EEG measurement implementation in the form of helmets may be possible with the use of only the BA 10 channels. Such implementation could suffice the data required for the advanced MCR application.

Furthermore, the average classification accuracy of the developed model from the combined use of the EEG and eye movement data was just 3% higher than the case of using the EEG data only (Table 6). Although the increase in classification accuracy is not significant from the use of additional eye movement data, the use of eye movement data may be important for human error reduction as looking at the right AOI is an important part of decision making by a nuclear operator.

While this study indicated the plausibility of using the EEG and eye movement data for attention monitoring based on mockup tasks with students as subjects, future study will consider using a full-scale simulator with professional reactor operators.

## 5. Conclusion

This study investigated the development of biosignal-based attention monitoring system for the purpose of preventing human error at NPP MCR. The system is based on a classification model for the presence or absence of operator attention. We designed general tasks and nuclear simulator tasks mimicking the situations in NPP MCR as the basis of model development. During these tasks, each subject's attention levels were examined and analyzed from their biosignals to develop the classification model. The biosignals used were the five frequency band EEG data and eye movement data. Through the use of the developed model, we demonstrated that the presence or absence of human attention can be classified with up to 90% in accuracy. The proposed methods could be adopted to other industrial applications for the purpose of human performance enhancement and/or human error reduction based on attention monitoring.

## Data Availability Statement

The original contributions presented in the study are included in the article/supplementary material, further inquiries can be directed to the corresponding author/s.

## Ethics Statement

The studies involving human participants were reviewed and approved by Korea Advanced Institute of Science and Technology (KAIST) Institutional Review Board (IRB) guideline. The patients/participants provided their written informed consent to participate in this study.

## Author Contributions

JK carried out the conception and design of study, acquisition of data, analysis and interpretation of data, and drafting of the manuscript. CK performed the acquisition, analysis, and interpretation of data. E-SJ was responsible for the design of an experiment and the drafting of the manuscript. M-SY supervised, interpreted data, and revised the manuscript critically for important intellectual content. All authors contributed to the article and approved the submitted version.

## Conflict of Interest

E-SJ was employed by company Samsung SDS. The remaining authors declare that the research was conducted in the absence of any commercial or financial relationships that could be construed as a potential conflict of interest.
